# Association between tobacco control policies and hospital admissions for acute myocardial infarction in Thailand, 2006-2017: A time series analysis

**DOI:** 10.1371/journal.pone.0242570

**Published:** 2020-12-02

**Authors:** Roengrudee Patanavanich, Stanton A. Glantz

**Affiliations:** 1 Center for Tobacco Control Research and Education, Department of Medicine, University of California San Francisco, San Francisco, California, United State of America; 2 Department of Community Medicine, Faculty of Medicine Ramathibodi Hospital, Mahidol University, Bangkok, Thailand; Universidad Adolfo Ibanez, CHILE

## Abstract

**Introduction:**

Studies in many countries have documented reductions of acute myocardial infarction (AMI) hospitalizations with smokefree policies. However, evidence on the association of cigarette tax with AMI events is unclear. There have been no studies of the associations between these two policies and AMI hospitalizations in Thailand.

**Methods:**

We used negative binomial time series analyses of AMI hospitalizations (ICD-10 codes I21.0-I21.9), stratified by sex and age groups, from October 2006 to September 2017 to determine whether there was a change in AMI hospitalizations as a result of the changes in cigarette prices and the implementation of a 100% smokefree law.

**Results:**

Cigarette price increases were associated with a significant 4.7% drop in AMI hospitalizations among adults younger than 45 (incidence rate ratio [IRR], 0.953; 95% confidence interval [CI], 0.914–0.993; p = 0.021). Implementation of the 100% smokefree law was followed by a significant 13.1% drop in AMI hospitalizations among adults younger than 45 (IRR, 0.869; 95% CI, 0.801–0.993; P = 0.001). There were not significant associations in older age groups.

**Conclusions:**

The Thai cigarette tax policy and the smokefree law were associated with reduced AMI hospitalizations among younger adults. To improve effectiveness of the policies, taxes should be high enough to increase cigarette price above inflation rates, making cigarettes less likely to be purchased; smokefree laws should be strictly enforced.

## Introduction

Cigarette smoking is a major risk factor of cardiovascular diseases (CVD) [1], with one of every three deaths from CVD attributable to smoking [2]. In Thailand, smoking causes nearly 250,000 hospitalizations for CVD and costs 10 billion THB (US$342 million) annually [3]. Cigarette smoking is also the most common risk factor of acute myocardial infarction (AMI) in patients younger than 45 years [4, 5]. Up to 92% of adults less than 45 years old with AMI are smokers, compared with only 40% of older patients [4, 6]. Smoking is also a predictor of coronary plaque rupture, particularly in young smokers [7, 8].

The World Health Organization (WHO) has recommended four evidence-based “best buy” interventions for tobacco use to reduce the economic impact of diseases caused by smoking, including tax increases, smokefree indoor workplaces and public places, health information and warnings, and bans on tobacco advertising, promotion, and sponsorship [9]. Thailand ratified the WHO Framework Convention on Tobacco Control (FCTC) in 2004 and since then has strengthened it tobacco control policies [10]. In 2019, WHO recognized Thailand’s high level of achievement in two of the best buy interventions: health warnings (since 2005) and smokefree policies (since 2010) [11].

Increasing cigarette taxes is an important intervention for reducing smoking prevalence [12–14], including in Thailand [15]. Thailand has increased its cigarette excise tax gradually since allowing imported cigarettes in 1991 [16]. The cigarette excise tax in Thailand increased from 55% to 90% of the ex-factory price (the price the manufacturer charges distributors) between 1992 and 2017. Driven at least in part by the increasing taxes, the price of a 20-cigarette pack of the most popular Thai brand (Krongthip) increased from 35 THB (US$1.1) in 1992 to 90 THB (US$2.8) in 2017 [17, 18] and smoking prevalence dropped from 32% in 1991 to 19% in 2017 [19].

The effects of smokefree laws on reducing AMI hospitalization have been well-documented, particularly among people younger than 65 years, in developed countries such as the U.S. and European countries [1]. Thailand began implementing smoking restrictions in 1992 and then repeatedly updated them to expand smokefree places [20]. In 2010, Thailand achieved 100% smokefree public places at the highest level of FCTC Article 8 (Protection of people from tobacco smoke) [20].

The ultimate goal of tobacco control policies is not to reduce smoking but to reduce the burden of disease and death caused by tobacco use. Numerous studies demonstrate the association of smokefree law implementation with the reduction of CVD morbidity and mortality [1]. However, evidence on the effects of cigarette tax on CVD events is limited [21]. A study in the U.S. found that an increase in the cigarette tax is associated with a significant decline in age-adjusted AMI hospitalization rates among men [22], but there are not yet studies in less-developed countries. To our knowledge, there are no studies in Thailand estimating the effects of raising cigarette excise taxes and smokefree policies on CVD events.

This study used negative binomial time series regressions to examine the statistical association between cigarette price increases (as the cigarette tax increased) and the strengthening the smokefree law from a partial ban to a 100% smokefree indoor workplaces and public places rule in 2010 [23–25] and AMI hospitalizations in Thailand between October 2006 and September 2017 and whether these associations varied across age and sex groups.

## Methods

We used the de-identified inpatient discharge data from the Universal Health Coverage Scheme, the largest public health insurance program in Thailand, which covers 75% of the Thai population [26, 27]. AMI was defined as a primary discharge diagnosis with International Classification of Disease version 10 (ICD-10) diagnostic codes I21.0-I21.9 [28]. These diagnostic codes include both ST elevation MI (STEMI) and non-ST elevation MI (NSTEMI). The monthly AMI hospitalizations were calculated from October 2006 to September 2017 (a total of 132 months) and stratified by sex and age groups (18–44, 45–59, and 60 years and over).

Data on cigarette prices were retrieved from the announcements of the Thai Excise Department and Revenue Department on the Royal Thai Government Gazette [29]. Market shares of cigarettes by brands were based on Excise Department data on the monthly taxed volume of cigarette packs by brand from October 2006 to September 2017 provided by the Action for Smoking and Health Foundation Thailand. Monthly average cigarette prices were translated into real THB as of September 2007 using the national consumer price index from the reports of the Thai Ministry of Commerce [30].

### Statistical analyses

We employed negative binomial time series regressions to analyze the 132-months of data from October 2006 through September 2017. (The data on the market share of cigarettes by brand from the Excise Department are only available through September 2017.) The dependent variable was number of monthly AMI hospitalizations, stratified on the sex and age (in separate analyses). The independent variables were the average cigarette price weighted by market share (in 10 THB) and a dummy variable to indicate the change of the smokefree policy, assigning 1 from June 2010 and 0 before that.

We controlled for the total monthly all-cause hospital admissions to account for the trend of monthly hospital uses [31]. We included dummy variables for month to allow for seasonal variation.

We also included a continuous time variable to allow for underlying secular trend (1 beginning on October 2006 and 132 on September 2017) and time squared (mean-centered) to allow for nonlinear effects in time. We included the quadratic term because in preliminary analysis (likelihood ratio tests and comparing R^2^) showed that including the quadratic time trend significantly improved the fit to the data [32].

Correlograms of the residuals revealed statistically significant autocorrelations in the residuals for the fit among all sex and age-specific outcomes except among men and women age 18–44. Therefore, we added a one-month lag of AMI hospitalizations as an independent variable to all the regression models (including the model for men and women ages 18–44 for consistency). Adding the lagged independent variable reduced all the autocorrelations to nearly zero and nowhere near significant (first lag autocorrelation for AMI among total population, 0.093; second, 0.021; and third, -0.004, with p-values 0.282, 0.544 and 0.748, respectively; results were similar for sex and age-specific outcomes). The lagged independent variables did not substantially affect the parameter estimates for the price and smokefree variables.

We analyzed the data using Stata 14 using the command *nbreg AMI CigPrice SmokeFree TotalAdmission i*.*MONTH TIME TIME*^*2*^ *l*.*AMI*, *irr* to estimate the predictive association for number of AMIs per month as a function of the independent variables described above. *i*.*MONTH* creates a set of dummy variables for the different months and *l*.*AMI* adds AMI’s lagged by 1 month as an independent variable. i*rr* reports results as incident rate ratios. The Stata documentation [33] provides more details on how the *nbreg* command relates to negative binomial model.

We also did the same analysis using a Poisson regression, which makes more assumptions about the data structure than negative binomial regression.

Ethics approval was not required because this study used deidentified data.

## Results

### Cigarette prices

During the study period there were four cigarette excise taxes increases: September 2007 (from 79% to 80% as a percentage of the ex-factory price) [34], May 2009 (from 80% to 85%) [35], August 2012 (from 85% to 87%) [36], and February 2016 (from 87% to 90%) [37]. We determined the weighted average cigarette prices based on different brand prices of the five most-sold brands in Thailand (Krongthip, Wonder, SMS, L&M, and Marlboro) and their monthly market shares. These five brands comprised over 90% of the total Thai market [38].

**Fig 1:**
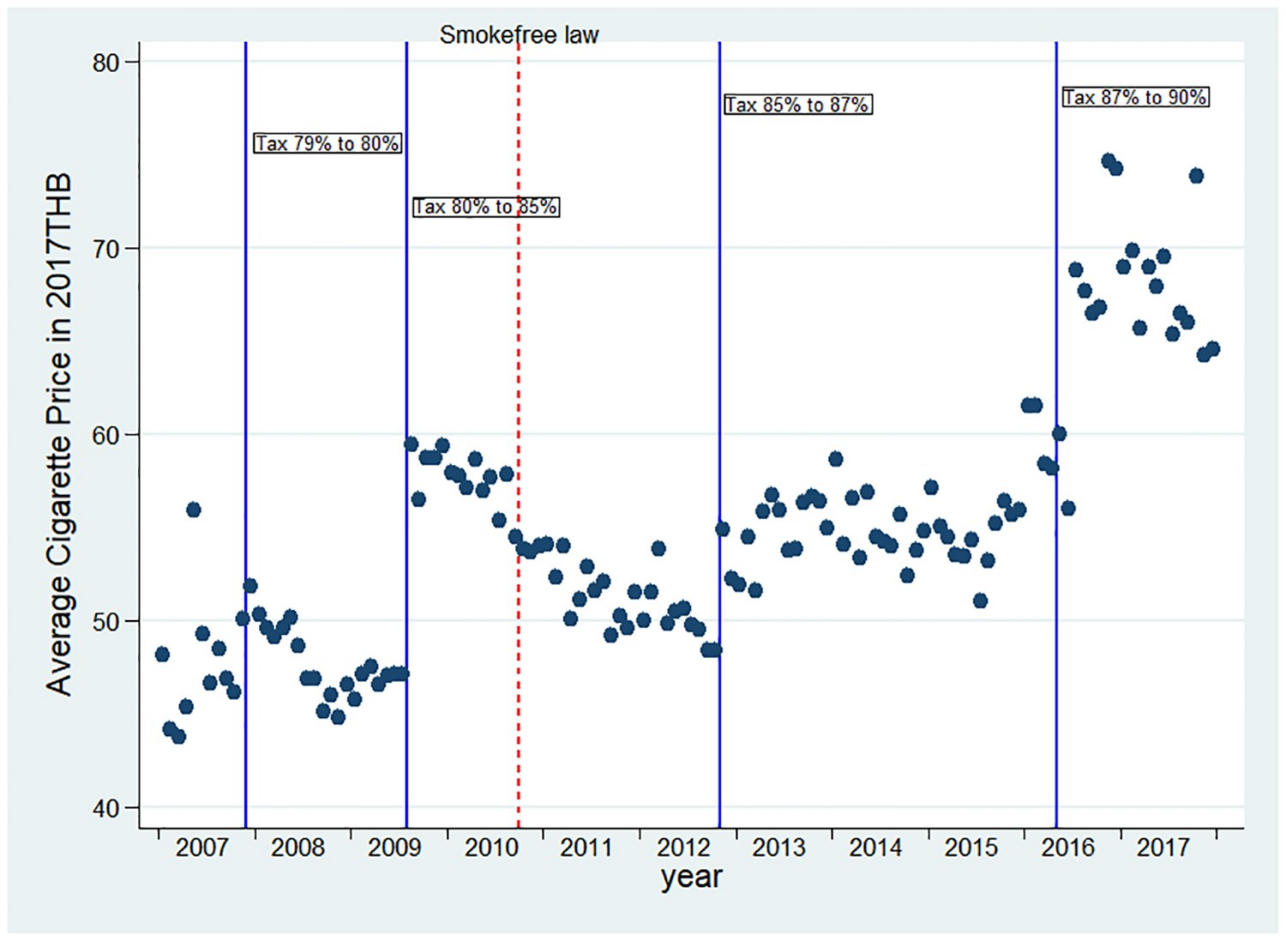
Average cigarette prices between 2006 and 2017. Dots are average cigarette price for each month. Vertical solid lines indicate the change in cigarette tax rates. The vertical dash line indicates implementation of Thailand’s 100% smokefree law. New low-priced cigarette brands and slim cigarettes introduced in 2010, made average cigarette prices in Thailand remain relatively unchanged or even lower during 2009–2016.

Real cigarette prices in Thailand did not uniformly increase over time (Fig 1), probably because of the introduction of new low-priced cigarette brands and slim cigarettes in Thailand in 2010 [35].

### Smokefree laws

As of October 2006, the smoking restriction law prohibited smoking in indoor air-conditioned places only [23, 24]. In June 2010, a 100% smokefree law was implemented that required all indoor public and work places and other open-air public places such as restaurants and markets to be smokefree [24] (Table 1).

**Table 1:** Smoke restrictions before and after June 2010 [39, 40].

Place	Oct 2006-May 2010	June 2010-September 2017
Health care facilities	Smoking was allowed in private rooms and designated areas.	All types of health care facilities were smokefree.
Educational facilities except universities	All schools were smokefree.	All schools were smokefree.
Universities	Smoking was allowed in private rooms and designated areas.	All areas inside buildings were smokefree. Smoking was allowed in designated areas only.
Government facilities	Smoking was allowed in private rooms and designated areas.	All areas inside buildings were smokefree. Smoking was allowed in designated outdoor areas only.
Indoor offices	Only air-conditioned workplaces were smokefree. Smoking was allowed in private rooms and designated areas.	All indoor offices were smokefree.
Restaurants	Only air-conditioned restaurants were smokefree.	All restaurants were smokefree.
Pubs and bars	No.	All pubs and bars were smokefree.
Public transportation	All areas were smokefree.	All areas were smokefree.

This was a substantial change because non air-conditioned restaurants and markets were more common in 2005 and only 33% of Thai people went to air-conditioned restaurants regularly [41].

### Associations of cigarette prices and smokefree laws on AMI

A total of 435,208 AMIs occurred between October 2006 and September 2017 in Thailand, 249,615 (57%) were males and 185,593 (43%) were females. Mean ages were 66 years; 64 years for males and 68 years for females. Average monthly AMI hospitalizations were 3,297. The proportions of AMI among age groups were 4.9% for age 18–44, 24.3% for age 45–59, and 68.2% for age 60 and older.

We found cigarette price increases and the smokefree laws were significantly associated with reduction of AMI hospitalizations among adults younger than 45 years, but not other age groups (Table 2). Cigarette price increases were associated with a significant 4.7% drop in AMI hospitalizations per 10 THB increase (incidence rate ratio [IRR], 0.953; 95% confidence interval [CI], 0.914–0.993; p = 0.021). Among 18–44 year olds, the point estimates were similar in men (IRR 0.948) and women (IRR 0.943), but the association was only statistically significant among men (Table 3).

**Table 2:** Associations between tobacco control policies and AMI hospitalizations stratified on age.

Variables	Total Population		Age 18–44		Age 45–59		Age 60 and up	
	IRR [95% CI]	p-value	IRR [95% CI]	p-value	IRR [95% CI]	p-value	IRR [95% CI]	p-value
Average Cigarette Price (per 10 THB)	0.999	0.903	**0.953**	**0.021**	1.008	0.534	0.995	0.671
	[0.978,1.020]		**[0.914,0.993]**		[0.983,1.034]		[0.970,1.020]	
100% Smoke-Free Law	1.004	0.841	**0.869**	**0.001**	0.978	0.364	1.023	0.271
	[0.966,1.044]		**[0.801,0.944]**		[0.932,1.026]		[0.977,1.071]	
Total admissions (in 1,000)	1.003	0.339	1.005	0.385	0.999	0.789	1.003	0.271
	[0.997,1.008]		[0.994,1.015]		[0.993,1.005]		[0.997,1.010]	
Time	**1.003**	**0.001**	**1.007**	**0.001**	**1.004**	**0.001**	**1.004**	**0.001**
	**[1.002,1.004]**		**[1.005,1.008]**		**[1.003,1.006]**		**[1.002,1.005]**	
Time2	**0.999**	**0.001**	0.999	0.318	**0.999**	**0.001**	**0.999**	**0.001**
	**[0.999,0.999]**		[0.999,1.000]		**[0.999,0.999]**		**[0.999,0.999]**	
Total number of AMIs	435,208		21,233		105,783		296,973	
Observations	131		131		131		131	
Pseudo R^2^	0.213		0.185		0.252		0.224	

Coefficients for monthly seasonal variables and the lagged variables not shown.

**Table 3:** Associations of tobacco control policies with AMI hospitalizations stratified by age and sex.

Variables	Male 18–44		Male 45–59		Male 60 and up		Female 18–44		Female 45–59		Female 60 and up	
	IRR [95% CI]	p-value	IRR [95% CI]	p-value	IRR [95% CI]	p-value	IRR [95% CI]	p-value	IRR [95% CI]	p-value	IRR [95% CI]	p-value
Average Cigarette Price (per 10 THB)	**0.948**	**0.026**	1.003	0.801	0.987	0.331	0.943	0.162	1.011	0.561	0.999	0.983
	**[0.904,0.994]**		[0.977,1.031]		[0.962,1.013]		[0.868,1.024]		[0.974,1.049]		[0.972,1.029]	
100% Smoke-Free Law	**0.861**	**0.002**	0.987	0.623	1.021	0.396	**0.836**	**0.030**	0.954	0.195	1.025	0.365
	**[0.784,0.947]**		[0.937,1.040]		[0.973,1.071]		**[0.712,0.982]**		[0.888,1.025]		[0.972,1.081]	
Total admissions (in 1,000)	0.997	0.646	0.998	0.463	1.003	0.327	**1.032**	**0.003**	1.001	0.811	1.004	0.318
	[0.986,1.009]		[0.991,1.004]		[0.997,1.010]		**[1.011,1.053]**		[0.992,1.010]		[0.997,1.011]	
Time	**1.009**	**0.001**	**1.006**	**0.001**	**1.004**	**0.001**	**1.005**	**0.001**	**1.004**	**0.001**	**1.004**	**0.001**
	**[1.007,1.010]**		**[1.004,1.007]**		**[1.003,1.006]**		**[1.002,1.007]**		**[1.003,1.005]**		**[1.002,1.005]**	
Time2	0.999	0.300	**0.999**	**0.001**	**0.999**	**0.001**	**1.001**	**0.794**	**0.999**	**0.002**	**0.999**	**0.001**
	[0.999,1.001]		**[0.999,0.999]**		**[0.999,0.999]**		**[0.999,1.001]**		**[0.999,0.999]**		**[0.999,0.999]**	
Number of AMIs	16,067		71,097		155,499		5,166		34,686		145,576	
Observations	131		131		131		131		131		131	
Pseudo R^2^	0.185		0.252		0.224		0.080		0.180		0.202	

Coefficients for monthly seasonal variables and the lagged variables not shown.

The implementation of the 100% smokefree law was followed by a significant 13.1% drop in AMI hospitalizations among adults aged 18–44 years (IRR, 0.869; 95% CI, 0.801–0.993; p = 0.001); the significant reduction of AMI hospitalizations among this age group was similar and significant in both sexes (Table 3).

The sensitivity analysis conducted with a Poisson regression (S1 Table) produced similar results as the main analysis using negative binomial regression (Table 2), except that the Poisson analysis showed smokefree laws associated with significantly more AMIs among people 60 years old and older. This unusual result may be due to the fact that the Poisson regression makes more assumptions about the underlying data structure than negative binomial regression.

## Discussion

This study highlighted the associations between raising cigarette prices and the implementation of a smokefree law on improving health. These two policies are population-based interventions to prevent smoking initiation, induce smokers to quit or reduce their cigarette consumption, and decrease exposure to secondhand smoke among non-smokers [42].

Several studies have indicated that smoking is the most common risk factor for AMI in young adults under age 45 [4, 6]. Our study demonstrates an association between increased cigarette prices and reduced AMI hospitalizations among this age group. This result is consistent with findings that younger adults were more responsive to changes in cigarette prices through increased taxes than older adults [12–14, 43]. Our finding that the significant associations with raising cigarette taxes were limited to males may reflect two factors. First, the number of male smokers under age 45 was 36 times higher than female smokers, and this difference declines with age [44]. Second, previous studies found AMI in young adults occurred 3 times more often in men than in women and heart disease developed 7 to 10 years later in women than in men [4, 45]. Similarly, our data showed the number of AMI hospitalizations was 3 times higher in men than in women at the age of 18–44 and the difference was smaller among older age groups. The fact that the point estimates were similar among men and women age 18–44 year olds (Table 3) suggests that the failure to reach statistical significance for the women may be due to the smaller number of AMIs among women.

The point estimates associated with raising cigarette prices on AMI hospitalizations were less than we expected. We did not find significant associations effects among the total population as observed in some other countries [46]. It could be the tax increases were not always followed by increase in cigarette prices because of industry price manipulation to buffer the price effect [47].

Consistent with previous studies [1], the implementation of the comprehensive smokefree law in Thailand was associated with a significant drop in AMI hospitalizations among young adults aged 18–44 years. The point estimate for the association of smokefree law on AMI hospitalizations was substantial, equivalent to about a 30 THB increase in price. It took about 10 years in Thailand to have a 30 THB increase in cigarette prices (the average cigarette price was 39 THB in 2006 did not increase to 67 THB until 2017). Many studies have also found that smoking bans have a greater effect on AMI occurrence among younger compared to older patients [48–51]. Other studies have shown that smoking bans have a greater effect on AMI hospitalizations among non-smokers compared to smokers due to reductions of secondhand smoke [52, 53]. Youth and young adults are exposed to higher rates of secondhand smoke than older adults, consistent with growing evidence that comprehensive smokefree laws contribute to a greater decrease in passive smoking than active smoking [48, 50, 52–54].

We did not see a significant drop in AMI hospitalizations in adults older than 45 years following the comprehensive smokefree law in Thailand. This could be due to ineffective law enforcement and compliance. The 2016 stakeholder’s assessment of Thailand’s compliance with the WHO FCTC Article 8 revealed that the level of implementation rated for effectiveness was low, especially the enforcement and public understanding of smokefree principles [20]. This assessment was consistent with the 2017 national smoking and drinking behavior survey data that showed a significant proportion of people experienced secondhand smoke in public places, especially marketplaces (74.5%), public transportation (68.2%), and restaurants (64.2%) [55].

Our findings indicated that if the price per pack of cigarettes increased by 10 THB, the number of AMI hospitalizations could be reduced by 4.7% among adults younger than 45 or save approximately 91 patients from having AMI annually (from our data, average monthly AMI hospitalizations among adults younger than 45 was 161). Additionally, the smokefree law could reduce the number of AMI hospitalizations by 13.1% among adults younger than 45 or save approximately 254 patients from having AMI annually. A previous study in Thailand showed an average hospital admission cost for a CVD patient was 43,000 THB (US$1,390) [3]. Thus, every 10 THB increase in the sale price of a pack of cigarette combined with implementation of the smokefree law could save approximately 345 AMI patients or 14.8 million THB (US$479,550) in hospital admission costs due to AMI annually. However, the direct medical cost for inpatient treatments only accounts for 9% of all costs attributable to smoking [56]. If other costs such as the direct medical cost for outpatient visits, indirect medical cost for transportation, out-of-pocket, and non-medical cost for loss of income for patients and caregivers and due to death were considered, preventing 345 patients from having AMI could save approximately 164.8 million THB (US$5.3 million).

**Fig 2:**
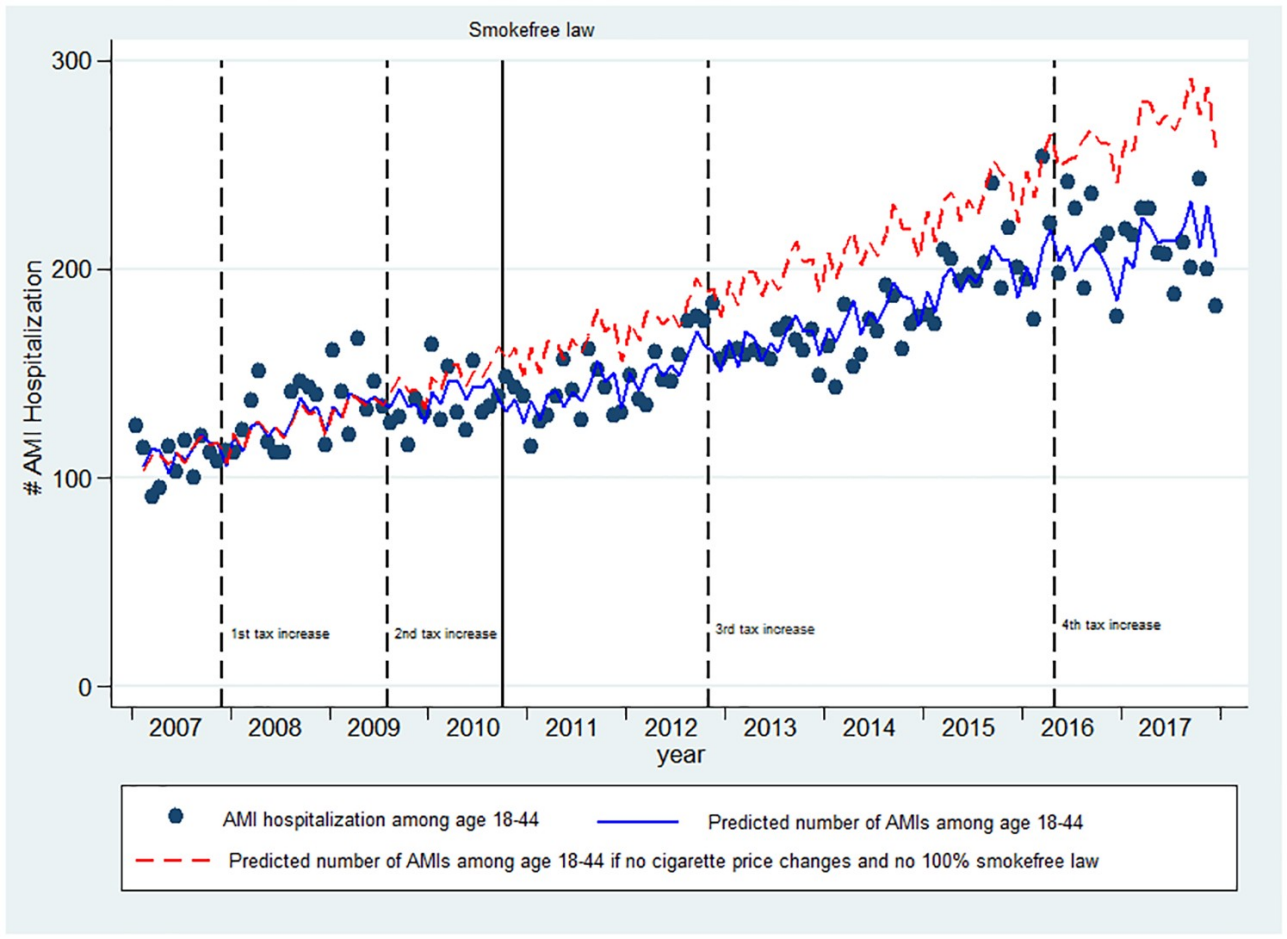
Association of increase in cigarette price and smokefree laws on AMI among young adults. The number of AMI hospitalizations among age 18–44 dropped significantly after the increase in cigarette price and the implementation of 100% smokefree law between October 2006 and September 2017. Solid lines (blue) on the plots are the predicted number of AMI hospitalizations per month from the negative binomial regression model. Dots are the number of AMI hospitalizations each month. Dashed lines (red) on the plots are the predicted numbers of AMI hospitalizations had there been no price changes and no 100% smokefree law. The ups and downs in the regression lines represent seasonal (monthly) variation in the number of AMI hospitalizations. Dash vertical lines indicate when the cigarette tax increased and a solid vertical line indicate when the 100% smokefree law was implemented.

The combined magnitude of the associations between price increases and smokefree law were substantial among 18–44 year olds. Between June 2010 and September 2017, the number of AMIs in this group were 3,190 below what would have been predicted absent these changes (Fig 2), accounting for a savings of at least 137 million THB (US $4.4 million) in direct health costs, which increased to 1.52 billion THB (US $49.2 million) when including the indirect health costs. The comparison of the rates of AMI hospitalizations by age groups had there been no price changes and no 100% smokefree law is shown in Table 4.

**Table 4:** Average monthly AMI hospitalization rates per 100,000 population by age groups with and without policy on cigarette tax and smokefree law.

Year	Age 18–44			Age 45–59			Age 60 and up		
	with policy	without policy	Difference	with policy	without policy	Difference	with policy	without policy	Difference
2006	0.39	0.38	2%	4.41	4.42	0%	20.80	20.76	0%
2007	0.41	0.41	0%	4.57	4.57	0%	21.04	21.05	0%
2008	0.47	0.46	1%	5.02	5.03	0%	23.47	23.46	0%
2009	0.50	0.51	-3%	5.39	5.36	1%	24.88	24.96	0%
2010	0.50	0.56	-12%	5.62	5.66	-1%	26.62	26.36	1%
2011	0.53	0.61	-17%	5.89	6.01	-2%	28.45	27.86	2%
2012	0.58	0.68	-17%	6.24	6.36	-2%	29.09	28.48	2%
2013	0.62	0.74	-19%	6.66	6.76	-2%	29.75	29.21	2%
2014	0.68	0.81	-19%	6.95	7.07	-2%	30.20	29.63	2%
2015	0.74	0.89	-19%	7.46	7.58	-2%	30.53	29.98	2%
2016	0.78	0.97	-26%	7.62	7.68	-1%	30.09	29.71	1%
2017	0.83	1.04	-26%	7.72	7.78	-1%	30.06	29.70	1%
2006–2017	0.59	0.67	-13%	6.13	6.19	-1%	27.08	26.76	1%

## Limitations

There are several limitations of this study. This study is an ecological study in which we do not have detailed information about individual patients, including their smoking status, so it is not possible to differentiate between events related to smoking or passive smoking. The study is based on hospitalization data, it could be possible that diagnosis coding was wrongly assigned and that there were variations in coding practice among hospitals [57]. Also, people under the Universal Health Coverage Scheme tend to have low socioeconomic status; therefore, our findings may not be generalized to people under other health insurance programs [26].

This study did not account for untaxed tobacco products such as hand-rolled cigarettes, which is a substitute for manufactured cigarettes in Thailand [34]. We did not explicitly consider any changes in air pollution such as PM_2.5_ due to the unavailability of the dataset for the entire country [58]. We did not include the health warning policy because Thai tobacco products have been compliant with the WHO FCTC’s health warning provisions since 2005 and the bans on tobacco advertising and promotion did not change during our study period [59, 60]. We did, however, include variables to account for seasonal variation and a secular trend to assume air pollution had not remained in a steady state throughout the period of the study.

Lastly, because cigarette excise tax policy in Thailand was changed in October 2017 from the ex-factory price to the retail selling price and from a one-tier to a two-tier ad valorem excise system with a minimum specific excise floor tax, we limited our analysis to prior this tax policy change [17].

## Conclusions

These findings support implementation of tobacco control policies in Thailand such as raising cigarette prices through increasing cigarette taxes and implementing the smokefree law. These laws not only decreased cigarette smoking prevalence, but also reduced smoking-caused diseases, notably AMI hospitalizations. To maintain effectiveness of tobacco control policies, the government should be wary of deceptive tobacco industry tactics that undermine the cigarette tax policy and should strengthen law enforcement of smokefree policies. Our results also suggest that improving implementation and enforcement of the smokefree law would further reduce AMI hospitalizations.

## Supporting information

S1 Table Association between tobacco control policies and AMI hospitalizations stratified on age (Poisson regression analysis). (PDF)

S1 Checklist (DOCX)
