# Supplementary material for: Association between tobacco control policies and hospital admissions for acute myocardial infarction in Thailand, 2006-2017: A time series analysis
Source: PLoS One. 2020 Dec 2;15(12):e0242570. doi: 10.1371/journal.pone.0242570 (PMC7710088; doi:10.1371/journal.pone.0242570)
Supplement: S1 Table — (PDF) [file pone.0242570.s001.pdf]

Association between tobacco control policies and hospital admissions for acute myocardial infarction in Thailand, 2006-2017: A time series analysis

Roengrudee Patanavanich and Stanton A. Glantz

S1 Table. Association between tobacco control policies and AMI hospitalizations stratified on age (Poisson regression analysis)

Variables	Total Population		Age 18-44		Age 45-59		Age 60 and up	
	IRR [95% CI]	p-value	IRR [95% CI]	p-value	IRR [95% CI]	p-value	IRR [95% CI]	p-value
Average Cigarette Price (in 10 THB)	1.001 [0.992-1.008]	0.942	**0.954 [0.920-0.989]**	**0.011**	1.009 [0.993-1.026]	0.252	0.997 [0.987-1.006]	0.484
100% Smoke-Free Law	1.007 [0.991-1.023]	0.413	**0.869 [0.807-0.937]**	**0.001**	0.98 [0.948-1.013]	0.233	**1.026 [1.006-1.046]**	**0.010**
#Total admissions (in 1,000)	1.001 [0.999-1.003]	0.146	1.004 [0.995-1.013]	0.372	0.998 [0.994-1.002]	0.405	1.002 [0.999-1.005]	0.069
Time	**1.003 [1.003-1.004]**	**0.001**	**1.007 [1.005-1.008]**	**0.001**	**1.004 [1.003-1.005]**	**0.001**	**1.004 [1.003-1.004]**	**0.001**
Time2	**0.999 [0.999-0.999]**	**0.001**	0.999 [0.999-1.000]	0.241	**0.999 [0.999-0.999]**	**0.001**	**0.999 [0.999-0.999]**	**0.001**
Total number of AMIs	435,208		21,233		105,783		296,973	
Observation	131		131		131		131	
Pseudo R2	0.927		0.457		0.826		0.903	
